# Biopreservative and Anti-Mycotoxigenic Potentials of *Lactobacillus paracasei* MG847589 and Its Bacteriocin in Soft White Cheese

**DOI:** 10.3390/toxins16020093

**Published:** 2024-02-07

**Authors:** Mohamed G. Shehata, Tawfiq Alsulami, Nourhan M. Abd El-Aziz, Hagar S. Abd-Rabou, Sobhy A. El Sohaimy, Amira M. G. Darwish, Karolina Hoppe, Hatem S. Ali, Ahmed Noah Badr

**Affiliations:** 1 Department of Food Technology, Arid Lands Cultivation Research Institute, City of Scientific Research and Technological Applications (SRTA-City), New Borg El-Arab 21934, Egypt; gamalsng@gmail.com (M.G.S.); nourhan.srta@gmail.com (N.M.A.E.-A.); elsohaimys@gmail.com (S.A.E.S.);; 2 Food Research Section, R&D Division, Abu Dhabi Agriculture and Food Safety Authority (ADAFSA), Abu Dhabi 20602, United Arab Emirates; 3 Food Science & Nutrition Department, College of Food and Agricultural Sciences, King Saud University, Riyadh 11451, Saudi Arabia; talsulami@ksu.edu.sa; 4 Department of Technology and Organization of Public Catering, Institute of Sport, Tourism, and Service, South Ural State University, 454080 Chelyabinsk, Russia; 5 Chemistry Department, Poznan University of Life Science, ul. Wojska Polskiego 75, 60-625 Poznan, Poland; karolina.gromadzka@up.poznan.pl; 6 Food Technology Department, National Research Centre, Cairo 12622, Egypt; hatem.owyean1@gmail.com; 7 Food Toxicology and Contaminants Department, National Research Centre, Cairo 12622, Egypt

**Keywords:** *Lactobacillus paracasei* MG847589, bacteriocin, biopreservation, anti-mycotoxigenic, soft white cheese, microstructure, shelf life, safe food production

## Abstract

Probiotics and their bacteriocins have increasingly attracted interest for their use as safe food preservatives. This study aimed to produce soft white cheese fortified with *Lacticaseibacillus* MG847589 (*Lb. paracasei* MG847589) and/or its bacteriocin; cheese with *Lacticaseibacillus* (CP), cheese with bacteriocin (CB), and cheese with both *Lacticaseibacillus* and bacteriocin (CPB) were compared to control cheese (CS) to evaluate their biopreservative and anti-mycotoxigenic potentials for prolonged shelf life and safe food applications. The effects of these fortifications on physiochemical, microbial, texture, microstructure, and sensory properties were studied. Fortification with *Lacticaseibacillus* (CP) increased acidity (0.61%) and microbial counts, which may make the microstructure porous, while CPB showed intact microstructure. The CPB showed the highest hardness value (3988.03 g), while the lowest was observed with CB (2525.73 g). Consequently, the sensory assessment reflected the panelists’ preference for CPB, which gained higher scores than the control (CS). Fortification with *Lb. paracasei* MG847589 and bacteriocin (CPB) showed inhibition effects against *S. aureus* from 6.52 log_10_ CFU/g at time zero to 2.10 log_10_ CFU/g at the end of storage, *A. parasiticus* (from 5.06 to 3.03 log_10_ CFU/g), and *P. chrysogenum* counts (from 5.11 to 2.86 log_10_ CFU/g). Additionally, CPB showed an anti-mycotoxigenic effect against aflatoxins AFB_1_ and AFM_1_, causing them to be decreased (69.63 ± 0.44% and 71.38 ± 0.75%, respectively). These potentials can extend shelf life and pave the way for more suggested food applications of safe food production by fortification with both *Lb. paracasei* MG847589 and its bacteriocin as biopreservatives and anti-mycotoxigenic.

## 1. Introduction

Food consumption is intended to deliver required nutrients, while functional foods provide additional properties that contribute positively to health, especially in preventing various diseases and disorders [1]. Increasing demands for natural and chemical-free products have led food research to search for an alternative technique for food biopreservation with novel strategies [2,3], and extending shelf life remains challenging [4]. The genus *Lactobacillus* is essential to modern food technologies for its potential to replace antibiotic growth promoters [5]. Various applications have recently been used to produce dairy products that resist mycotoxicological contamination and can reduce dairy product contamination [3,6,7]. The antibacterial efficacy of *Lactobacillus* and its bacteriocin (ribosomal peptides or proteins synthesized by bacteria) is a promising alternative to natural preservatives that prevent or reduce the growth of foodborne pathogen *S. aureus* [8,9,10]. Furthermore, *Lactobacillus* bacteria suppressed the conidial germination and mycelial growth of *Aspergillus parasiticus* and *Penicillium chrysogenum*. There are opportunities for future research to prevent fungal growth and eliminate mycotoxins from food or their transformation into less dangerous compounds using the strains of lactic acid bacteria [11,12].

Natural contaminants such as mycotoxins, are a significant food safety concern, considered the main hazard in food products, particularly aflatoxins (AFB_1_ and AFM_1_) classified in Group 1 (human carcinogen) by the International Agency for Research on Cancer [13]. Several applications were recorded by efficiently reducing the aflatoxin contamination using antagonism impact [14]. Otherwise, the application of natural extracts rich in bioactive molecules can reduce these types of hazards [15,16]. In addition to antifungal potentials, the *Lactobacillus* bacterial strain showed many anti-mycotoxigenic possibilities to be widely used in food and feed commodities to either inhibit the production of mycotoxins or reduce the quantity of already produced mycotoxins through physical and chemical binding involving the use of acidification and absorbents with a multi-mycotoxin binding capacity [17]. White cheese is the dominating category and popular choice, with approximately 32% of the cheese market in Egypt [18]; therefore, it can be considered the perfect cheese product for producing probiotic cheese as a delivery system for viable probiotic microorganisms.

Additionally, the consumption of probiotic cheese has been found to attenuate exercise-induced immune suppression, improve symptoms of constipation, and improve body mass index and blood pressure indices [19]. The shelf life of white cheese was reportedly found to be between days 14 and 28 as white cheese generally ages slowly, while the microbiota agents can potentially prolong cheese shelf life [20]. However, some investigations focused on the metabolomic benefits of other milk sources [21].

Nevertheless, cheese manufacturing is carried out through several steps, including ripening, storage, and handling, and several issues could occur, such as microbial contamination. A novel strain of *Lacticaseibacillus* MG847589 (*Lb. paracasei* MG847589), isolated in previous work from local dairy products, has a bioactive metabolite (bacteriocin) that has a potential application in cheese production. This study aimed to produce soft white cheese fortified with this strain (*Lb. paracasei* MG847589), its bacteriocin, and their combination to evaluate their biopreservative and anti-mycotoxigenic potentials for prolonged shelf life and safe food applications. Also, this study aimed to evaluate this strain’s functionality to improve cheese products’ safety and preservation qualities, such as reducing contamination levels with fungi that produce mycotoxins. The effects of these fortifications on physiochemical, microbial, texture, microstructure, and sensory properties were studied.

## 2. Results

### 2.1. Physicochemical Characteristics of Functional White Cheese

Changes in the mean values of moisture, protein, fat, and fiber in dry matter (DM) are presented in (Table 1). All parameters ranged in levels usually observed in soft white cheeses [22,23]. All the cheese treatments did not affect the moisture, total protein, fiber, and fat content. These results agree with previous studies in which various adjunct cultures were used in white cheeses [24,25].

The pH and lactic acid were found at levels usually observed in soft white cheeses [26,27]. In general, soft white cheese production targets high acidification rates using starter cultures that can differ among producers or areas of milk origin [28]. It was observed that *Lb. paracasei* and bacteriocin did not significantly affect the chemical composition of the cheese studied, except for the acidity values that were significantly higher in the presence of the probiotic *Lb. paracasei* MG847589 treatments: CP and CPB. A similar observation was reported by Allam et al. [5].

**Figure 1:**
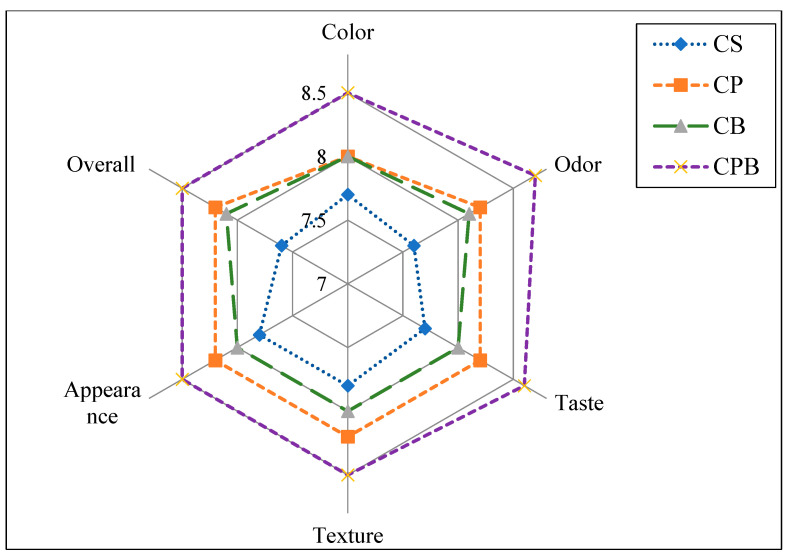
Sensory assessment of prepared cheese. Control cheese (CS); Probiotic cheese with strain MG847589 (CP); Cheese with bacteriocin (CB); Cheese with probiotics and their bacteriocin (CPB). Data represented are mean of triplicates ± SD.

The sensory assessment of soft white cheese products is shown in Figure 1. All sensory evaluation parameters were affected by and reflected panelists’ preference for CPB, followed by CP and CB. These results are correlated with texture analyses and indicated that increased hardness of the products fortified with probiotics or bacteriocin positively affected their sensory properties. The enhanced microstructure of CPB pronounced in (Figure 1) was reflected in texture scores. Sensory perception of innovative products is crucial as it is one of the keys to the widespread flavorful and wholesome image that dairy foods continue to enjoy with the consumer. Consequently, sensory measurement is often the final step in many experiments or applications for quality or consistency evaluation [29].

**Figure 2:**
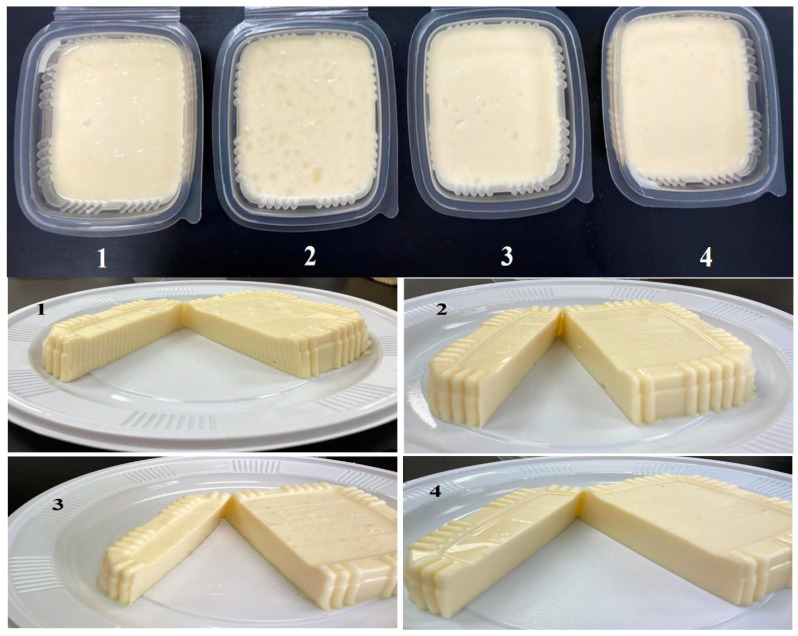
The morphology of manufactured cheese using several strategies. 1: Control cheese (CS); 2: Probiotic MG847589 cheese (CP); 3: Cheese with bacteriocin (CB); 4: Cheese with probiotics and their bacteriocin (CPB).

Color analyses indicated that compared with control cheese, cheese with probiotics (CP), bacteriocin (CB), and probiotics and bacteriocin (CPB) did not significantly affect cheese lightness (L), yellowness (b), or redness (a). However, CP tended to be slightly yellowish, as shown in (Figure 1), exhibiting soft white cheese products. Sensory properties illustrated in Figure 2 showed that CPB color was preferable. Similar observations were recorded for probiotic cheese applying two *lactobacilli* strains [30].

### 2.2. Microbiological Analysis of Cheese during Maturation and Storage

Microbiological analyses of the cheese samples were carried out during cold storage for different microbial groups when fresh (1 day) and after 15, 30, and 45 days (Table 2). Fortification with the probiotic strain, bacteriocin, or their mixture affected the *Lactobacilli* counts significantly (*p* < 0.05) compared to the control samples. In all cheese samples, coliforms, yeasts, and mold were not detected during storage except on the 30th and 45th day of storage for control and the 45th day of storage for probiotic treatment. Adjunct probiotic cultures were reported to have the ability to reduce coliforms during cheese maturation faster than in cheeses produced with a single starter culture [31,32,33].

In Table 2, the counts of cocci did not significantly differ among all samples during cheese storage. On the other hand, the addition of probiotics significantly increased the population of *Lactobacilli* (*p* < 0.05) along with providing a healthy character to the cheese samples since the *Lactobacilli* population was maintained at high levels (>10.6 log_10_ CFU/g) [34] during 45 days of storage. The cheese with probiotics and bacteriocin (CPB) significantly affected the *Lactobacilli* counts in cheese (8.42 to 7.46 log_10_ CFU/g) compared to the cheese with probiotics (CP) (8.17 to 7.60 log_10_ CFU/g).

*Lactobacilli* counts most likely originated from starter and probiotic cultures but also from milk non-starter cultures that survived after pasteurization [35]. The decreased number of lactobacilli during ripening and storage may be due to low pH, high salt content, lack of fermentative sugars, or possible bacteriocin production.

### 2.3. Texture Profile Analyses (TPA)

Texture profile analyses of functional soft white cheese are illustrated in Table 3. Comparing the three treatments with control (CS), the results showed that the highest hardness values were observed with CPB, followed by CP, CS, and then CB (3988.03, 3357.73, 2648.73, 2525.7 g, respectively) in cycle one. CP treatment showed higher adhesive force, adhesiveness, and springiness (378.17 g, 378.17 mJ, and 6.71 mm, respectively). Applying bacteriocin in CB significantly decreased the hardness of cycle 1 and ycle 2 (2525.73 g and 2016.03 g, respectively). The reduction in hardness in soft cheese with bacteriocin may be related to moisture content (64.87%), which acts as a plasticizer in the protein matrix. A similar observation was reported by Zaky and Mahmoud [4].

### 2.4. Microstructure of Cheese Samples

**Figure 3:**
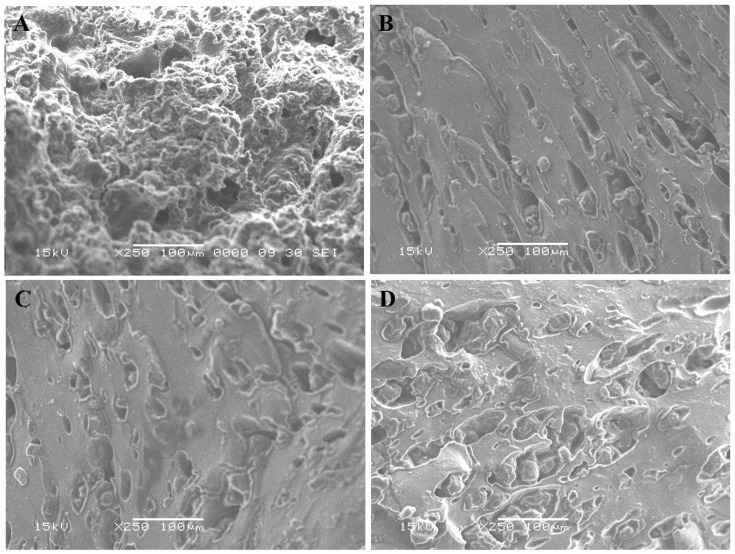
Cross-section of cheese samples captured using the SEM. (**A**): Control cheese (CS); (**B**): A probiotic MG847589 cheese (CP); (**C**): Cheese with bacteriocin (CB); (**D**): Cheese with probiotics and their bacteriocin (CPB).

Scanning electron micrographs of the cross-section in soft white cheese products are presented in Figure 3. Compared to control soft white cheese (Figure 3A), cheese with *Lb*. *paracasei* (CP) (Figure 3B) showed a porous structure that may be reflected in texture analyses showing the highest adhesiveness (Table 3). Fewer pores were observed in CB (Figure 3C), and the smooth structure reflected less hardness (Table 3). Cheese with probiotics and bacteriocin (CPB) (Figure 3C) showed an intact structure, as low moisture and high acidity might cause the highest hardness and adhesive force (Table 3). Microstructure differences were reflected significantly in the panelist’s evaluation to prefer CPB hard texture (Figure 1). These observations were noticed as well in the appearance of soft white cheese products (Figure 2). Application of probiotics, bacteriocin, or their mixture to soft cheese is recommended for the maintenance of sensory properties in addition to microbiological safety [4].

### 2.5. Inhibitory Effects of Lb. paracasei MG847589 against Pathogenic Microorganisms

**Figure 4:**
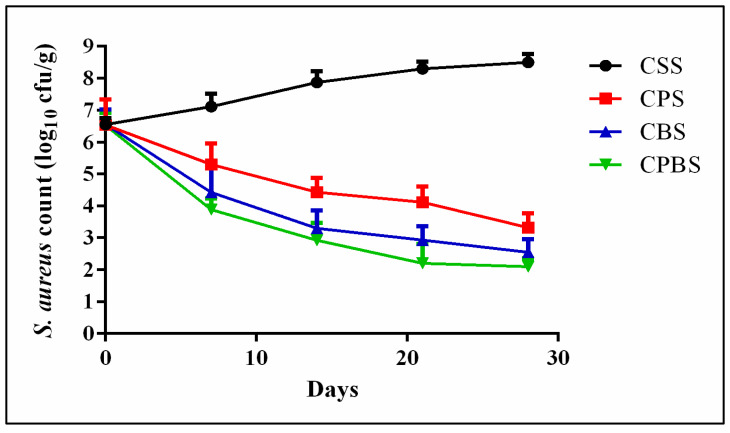
Inhibition rate of *S. aureus* in soft white cheese products throughout 28 days of storage at 4 °C. *S. aureus* count is expressed as mean values ± SD (SD: standard deviation; *n* = 3; *p* ≤ 0.05). Control cheese with commercial starter (CSS); Cheese with probiotic lactic acid bacteria *Lb. paracasei* MG847589 (CPS); Cheese with bacteriocin (CBS); Cheese with probiotics and their bacteriocin (CPBS).

The inhibition effects caused by *Lb. paracasei* MG847589 against *S. aureus* are shown in (Figure 4). The cheese fortification with *Lb. paracasei* MG847589 (CPS) showed an inhibition effect against *S. aureus*, decreasing its colonies from 6.54 to 3.32 log_10_ CFU/g after 28 days of storage (*p* > 0.05); also, the cheese fortification with *Lb. paracasei* MG847589 and bacteriocin (CPBS) showed an inhibition effect against *S. aureus*, from 6.52 to 2.10 log_10_ CFU/g after 28 days of storage (*p* > 0.05). *L. casei* subsp. *paracasei* was reported to exhibit inhibition effects, at the rates of 7.87% and 23.63%, against *S. aureus* on the 14th and 21st day of storage, respectively [36].

### 2.6. Inhibitory Effect of Lb. paracasei MG847589 against Pathogenic Bacteria

The inhibition effects caused by *Lb. paracasei* MG847589 against *S. aureus* are shown in (Figure 4). The cheese fortification with *Lb. paracasei* MG847589 (CPS) showed an inhibition effect against *S. aureus*, decreasing its colonies from 6.54 to 3.32 log_10_ CFU/g after 28 days of storage (*p* > 0.05); also, the cheese fortification with *Lb. paracasei* MG847589 and bacteriocin (CPBS) showed an inhibition effect against *S. aureus*, from 6.52 to 2.10 log_10_ CFU/g after 28 days of storage (*p* > 0.05). *L. casei* subsp. *paracasei* was reported to exhibit inhibition effects, at the rates of 7.87% and 23.63%, against *S. aureus* on the 14th and 21st day of storage, respectively [36].

**Figure 5:**
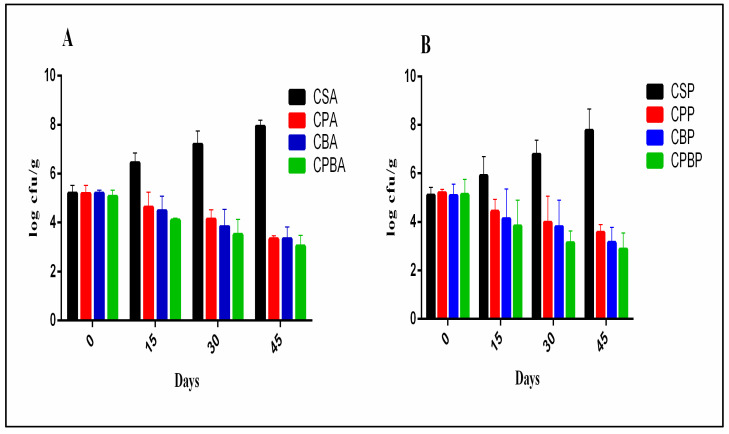
Inhibition of *A. parasiticus* and *P. chrysogenum* in soft cheese products during 45 days of storage at 4 °C. Inhibition rate expressed as mean ± SD (SD: standard deviation; *n* = 3; *p* ≤ 0.05). (**A**) *A. parasiticus*: Control cheese with commercial starter (CSA); Cheese with probiotic *Lb. paracasei* MG847589 (CPA); Cheese with bacteriocin (CBA), Cheese with probiotics and their bacteriocin (CPBA). (**B**) *P. chrysogenum*: Control cheese with commercial starter (CSP); Cheese with probiotic *Lb. paracasei* MG847589 (CPP); Cheese with bacteriocin (CBP), Cheese with probiotics and their bacteriocin (CPBP).

The presence of *Lb. paracasei* MG847589 in CPA and CPP treatments succeeded in decreasing the *A. parasiticus* and *P. chrysogenum* counts from 5.18 to 3.33 and 5.20 to 3.55 log_10_ CFU/g, respectively, after 45 days of storage (*p* > 0.05), indicating that the probiotic culture had an inhibitory effect against these fungal pathogens (Figure 5). After 45 days of storage, *A. parasiticus* and *P. chrysogenum* counts decreased from 5.06 to 3.03 and 5.11 to 2.86 log_10_ CFU/g in treatments CPBA and CPBP (*Lb. paracasei* MG847589 + bacteriocin), respectively (Figure 5). The ability of *Lb. paracasei* to inhibit *A. parasiticus* ITEM11 was reported by Shehata et al. [7]. The observed reduction in food pathogens in formulations fortified with *Lb. paracasei* MG847589 or its bacteriocin, compared to the negative control after 45 days of storage, can be relied on for the production of a series of antimicrobial compounds such as lactic acid, organic acids, hydrogen peroxide, ethanol, and diacetyl, which can inhibit pathogenic bacteria and fungi.

Furthermore, this strain can produce bacteriocin with a molecular weight of 2611 Da and peptides that show anti-Gram-positive and anti-Gram-negative bactericidal activity [7,37]. Consequently, probiotic strains that exhibit antimicrobial activity against spoilage or pathogenic bacteria within the matrix in which they are incorporated represent an interest for industrial application, as in addition to performing their probiotic effects, they contribute to extended products’ shelf life [38,39].

### 2.7. Antimycotoxigenic Effect of L. paracasei MG847589

The impact of applied treatment in manufactured cheese was also evaluated for the detoxification effect since AFM_1_ contaminated the raw materials or when the cheese samples were exposed to cross-contaminated by AFB_1_, as shown in Table 4 and Table 5. The result exhibited that, the increment in incubation time for the exposed spiked toxin to cheese treated by probiotic, its metabolite bacteriocin, or their mixture reflected increased detoxification potency (Table 4). The degradation ratio in AFM_1_-contaminated samples was recorded more efficiently than the reduction reported for the AFB_1_-spiked samples. After 48 hrs of incubation of the toxin within probiotic, bacteriocin, or their mixture, the detoxification ratio spanned between 63% and 69% for the AFB_1_ contamination, and between 64% and 71% for the AFM_1_-spiked in the cheese samples.

Previous studies referred to the better impact of bacteriocin as a probiotic metabolite to access aflatoxin detoxification [9,37,40]. Moreover, it was reported that several probiotics can reduce aflatoxin contamination through various mechanisms [41,42]. The results reflected the uniqueness of the applied strain to possess a detoxification potency, represented by the so-close efficiency of the bacterial cells and their metabolite bacteriocin. These results indicate the possibility of utilizing *L. paracasei* as a common starter in the predicted contaminated raw materials, which may be used for fresh or semi-fresh products; this step will provide an additive characteristic regarding the safety of the final dairy product.

Bacterial metabolites, particularly those generated by probiotic bacteria, can potentially contribute to the decontamination of aflatoxins via numerous approaches. The results exhibit variations in applying entire bacteria or metabolites in the targeted products [17,40]. Introducing bacterial cells into food items was crucial in influencing mycotoxicological fungi’s development and inhibiting mycotoxins’ formation. Certain beneficial bacteria can outcompete fungi that produce aflatoxin to acquire nutrients and occupy physical space. Through the process of colonizing similar ecological niches, these bacteria can restrict the development and propagation of toxin-producing fungi, resulting in a reduction in aflatoxin contamination [43,44].

The abovementioned phenomenon is often referred to as competitive exclusion. The second mechanism could be linked to the antagonism phenomena. Certain bacterial species can synthesize compounds with antifungal characteristics, impeding fungi proliferation that creates aflatoxins [45]. The potential impact of these metabolites includes the disruption of fungal cell membranes, interference with their metabolic activities, and the production of enzymes that break down aflatoxins [46,47].

Several bacterial species have been shown to exhibit enzymes that can degrade aflatoxins into molecules that are either less toxic or non-toxic [48]. The enzymatic activity can mitigate the toxicity of food and feed items that have been contaminated. It is plausible that beneficial bacteria have enzyme pathways capable of altering aflatoxins into less harmful variants or eliminating their toxicity [49]. These routes could be used to improve the safety of food and feed products. Specific bacterial metabolites can potentially adsorb aflatoxins, forming a binding interaction that hinders their absorption in vitro [12,41] or in vivo inside the gastrointestinal tracts of animals or humans [33,47]. The study consistently identifies certain strains of bacteria and their metabolites that can decrease aflatoxin exposure successfully. Nevertheless, it is crucial to acknowledge that the effectiveness of using bacterial metabolites for aflatoxin decontamination may differ depending on several aspects, including the particular bacterial strains used, environmental circumstances, and the extent of aflatoxin contamination.

## 3. Conclusions

Fortification with *Lb. paracasei* MG847589 increased acidity and microbial counts, which may affect the porous microstructure, while bacteriocin enhanced the microstructure to be intact. CPB showed a hard texture, while CB tended to be softer. Consequently, the sensory assessment reflected the panelists’ preference for CPB, which gained higher scores than the control (CS). Fortification with *Lb. paracasei* MG847589 and bacteriocin (CPB) showed inhibition effects against *S. aureus, A. parasiticus*, and *P. chrysogenum*,—as reflected by their reduced counts—which indicates their preservative potentials. Additionally, CPB showed significant anti-mycotoxigenic effects against aflatoxin B_1_ and M_1_. These potentials can extend shelf life, guarantee food safety, and encourage recommendations for fortification with both *Lb. paracasei* MG847589 and its bacteriocin as biopreservatives for many food applications.

## 4. Materials and Methods

### 4.1. Materials and Microorganisms

*Lactobacillus paracasei* MG847589 [GenBank accession No. MG847589] was isolated from traditional Egyptian Karish cheese [7]. The strain is currently preserved at −80 °C in 20% glycerol. Before inoculation, the strain was activated in de Man Rogosa and Sharpe (MRS) broth (37 °C/24 h). The commercial rennet enzyme and commercial starter culture Yo-Mix 495 were gifted by Dairy Pilot Plant, Alexandria University, Egypt. The milk protein (MPC), milk powder (RCM), and butter were purchased from the local market. Bacteriocin of the bacteria was extracted and purified as described before [7].

### 4.2. White Cheese Preparation

White cheese was manufactured using the technique suggested by Tamime et al. [50], albeit with some modifications (Figure S1). Target total solids were 38%, 29% protein, and 7% fat content in the standardized reconstituted milk. A laboratory homogenizer was utilized for the MPC and RCM blinding in water (20965 g force/6 min). The resultant was stood to age overnight (4 °C) to ensure that powders were evenly dispersed before pasteurization.

The mixture was divided into three sections, each with a different type of cheese: a control cheese with commercial starter (CS, 1.81 × 10^9^ CFU/mL); a probiotic cheese (CP, 1.34 × 10^9^ CFU/mL) of *L. paracasei* MG847589; and a bacteriocin-supplemented cheese (CB, at 500 AU/mL). The fourth portion was a combination of probiotics and bacteriocin (CPB). The commercial starter (Yo-Mix 495) containing *S. thermophilus* and *L. delbrueckii* was re-activated in milk before being added to the mixture. The cheeses were then mixed and left undisturbed for two hours. Table 6 shows the ingredients for producing white cheese (1 Kg).

### 4.3. Physicochemical Analysis

The pH value of all the cheese samples produced was measured by immersing the electrode of a digital pH meter (ADWA AD1030, Inc., Romania) directly into the cheese samples. The titratable acidity (expressed as lactic acid per 100 g of cheese) was determined. The moisture content was determined by drying 5-gram samples in an oven (70 °C/24 h), while the fat and fiber contents were determined following AOAC protocol [50]. The total nitrogen (TN) was determined following the Kjeldahl procedure [51] and was expressed as crude protein on a dry weight basis.

A tristimulus colorimeter (Smart Color Pro, USA) was utilized to determine the samples’ color characteristics. The color was measured using L, a, and b values, where L values range from 0 (black) to 100 (white), where positive values indicate redness, negative a values indicate greenness, positive b values indicate yellowness, and negative b values indicate blueness. The color analysis was conducted in triplicate, and the means ± SD were recorded.

### 4.4. Microbiological Profile Analysis of Cheese

Representative samples of cheese weighing 10 g were analyzed at various time intervals (1st, 7th, 15th, 30th, and 45th days) throughout the storage period. The samples were blended with 90 mL of sterile saline (0.9% *w*/*v*) solution. Microbiological tests for total aerobic mesophilic bacteria, *Lactobacilli* count, *S. thermophiles*, yeasts, and molds were performed according to the previous methodology [52,53]. All cell counts were expressed as log_10_ CFU/g of cheese.

### 4.5. Texture Profile Analyses (TPA)

The texture profile analysis (TPA) was carried out using a texture analyzer (TA1000, Lab Pro (FTC TMS-Pro), USA) following the method proposed before [54]. The TPA parameters, including peak force of the first compression (hardness cycle 1) (g), peak force of the second compression (hardness cycle 2) (g), adhesive forces, adhesiveness, resilience, springiness, and springiness index, were determined from force–time curves [55]. Texture profile analyses (TPA) were carried out in triplicates on day one [56].

### 4.6. Scanning Electron Microscopy and Sensory Evaluation

The cheese samples were prepared and fixed using glutaraldehyde solution (3%) as described before [57]. Panelists (a group of 20 humans) conducted a sensory evaluation of cheese, as Allam et al. [58] described. Sensory evaluation was conducted following institutional committee approval. The samples’ color, odor, taste, texture, appearance, and overall acceptability were evaluated using a scale of ten categories ranging from 1 (dislike) to 9 (like). For the scanning electron microscopy (SEM) inspection, samples were first given a sputter coating of gold ions using an Edwards model S 140A sputter coater to create a conducting medium. Sputtered materials were then scanned using a scanning electron microscope (SEM) with a JEOL Model JSM-T20.

### 4.7. Antimicrobial Assessment against Food Pathogens

Approximately 100 g of cheese was divided into sterile plastic bottles (200 mL). Cheese samples were divided into four treatments for each pathogen. Following previous work, probiotic bacteria were inoculated (1 mL/100 g cheese) to provide a system containing 7 log_10_ CFU/g of probiotic strain [7,59,60]. For pathogens, 6.5 log_10_ CFU/g of *S. aureus*, 5 log_10_ CFU/g of *A. parasiticus* ITEM 698, and 5 log_10_ CFU/g of *P. chrysogenum* ATCC 11709 were inoculated individually. Pathogen treatment groups are illustrated in (Table 5). Following inoculation, the electric mixer (Kenwood, UK) was used to shake all cheese samples (5 min). Afterward, they were stored (at 6 °C/45 days), resulting in 48 samples (3 pathogenic strains x 4 treatments x 4 storage time intervals). Viable cell counts were performed on each sample at 0, 15, 30, and 45 days of refrigerated storage. For the viable cell counts of fungi strains, potato dextrose agar (Sigma Aldrich, St. Louis, MO, USA) was used for 48 h/25 °C. For *S. aureus*, mannitol-sodium chloride-phenol red agar (Merck, Lowe, NJ, USA) was used for 24 h/37 °C. The results were expressed as means of log_10_ CFU/g cheese.

### 4.8. Anti-Mycotoxigenic Assessment against Aflatoxins (AFB_1_ and AFM_1_)

Certified vials of the AFB_1_ and AFM_1_ were utilized for spiked cheese (Sigma-Aldrich). The standards were dissolved in phosphate buffer saline (PBS, 400 ng/mL) and spiked in the targeted samples. The biopreservative activity of the MG847589 strain was estimated using white cheese as a food model. Samples were randomly assigned to one of four treatments, where different amounts of aflatoxins were applied (Table 5). The bacterial effectiveness and bacteriocin in reducing aflatoxin content were investigated against a control.

Quantitative determination of AFs was conducted using the Agilent 1100 HPLC system. The mobile phase was methanol (1): acetonitrile (3): and water (6). The determination was achieved using the previously mentioned conditions [61].

### 4.9. Statistical Analysis

The experiments were performed in triplicates and expressed in mean ± SD. The ANOVA with a general linear model was used to test for significance, and *p*-values of less than 0.05 were considered significant (using SPSS Ver.20).

## Figures and Tables

**Table 1:** Physicochemical analyses of functional soft white cheese.

Parameters	CS	CP	CB	CPB
**Chemical composition**				
**Moisture** **(%)**	64.16 ± 1.20 ^a^	62.50 ± 0.50 ^a^	64.87 ± 2.07 ^a^	62.55 ± 1.42 ^a^
**Protein** **(%)**	8.41 ± 0.31 ^a^	8.51 ± 0.07 ^a^	8.40 ± 0.25 ^a^	8.39 ± 0.18 ^a^
**Fat** **(%)**	6.65 ± 0.26 ^a^	6.46 ± 0.35 ^a^	6.31 ± 0.46 ^a^	6.62 ± 0.45 ^a^
**Fiber** **(%)**	0.50 ± 0.02 ^a^	0.55 ± 0.05 ^a^	0.54 ± 0.06 ^a^	0.53 ± 0.03 ^a^
**pH**	6.21 ± 0.11 ^a^	6.01 ± 0.35 ^a^	6.20 ± 0.22 ^a^	5.95 ± 0.21 ^a^
**Acidity** (g lactic acid/g)	0.53 ± 0.01 ^c^	0.61 ± 0.02 ^ab^	0.58 ± 0.01 ^b^	0.65 ± 0.02 ^a^
**Color analysis**				
L*	90.20 ± 1.33 ^a^	90.83 ± 1.16 ^a,b^	92.48 ± 0.74 ^b^	91.16 ± 1.76 ^a,b^
a*	−0.37 ± 0.07 ^a^	−0.42 ± 0.03 ^a^	−0.42 ± 0.02 ^a^	−0.41 ± 0.03 ^a^
b*	6.46 ± 0.25 ^a^	7.23 ± 1.26 ^a^	6.60 ± 0.20 ^a^	6.20 ± 0.60 ^a^

The different letters in superscripts next to the mean values in the same rows within variables indicate significant differences (*p* ≤ 0.05). Control cheese with commercial starter (CS); Cheese with probiotic lactic acid bacteria *Lb. paracasei* MG847589 (CP); Cheese with bacteriocin (CB); Cheese with probiotics and its bacteriocin (CPB).

**Table 2:** Microbiological evaluation (CFU/mL) of cheese samples during ripening and storage.

Cheese	Time (Days)	Total Aerobic Counts	*Lactobacilli*	*Cocci*	*Coliforms*	*Yeasts* and Mold
CS	1	6.51 ± 0.14 ^ef^	7.74 ± 0.47 ^abcde^	7.64 ± 0.36 ^ab^	ND	ND
	15	6.44 ± 0.16 ^f^	7.60 ± 0.21 ^bcde^	7.65 ± 0.31 ^ab^	ND	ND
	30	7.00 ± 0.20 ^cdef^	7.06 ± 0.24 ^ef^	7.43 ± 0.36 ^abcd^	2.36 ± 0.23 ^b^	3.54 ± 0.39 ^b^
	45	8.07 ± 0.13 ^ab^	6.40 ± 0.28 ^f^	6.13 ± 0.09 ^d^	3.31 ± 0.22 ^a^	4.11 ± 0.16 ^a^
CP	1	6.93 ± 0.54 ^cdef^	8.17 ± 0.34 ^abc^	7.60 ± 0.48 ^abc^	ND	ND
	15	7.26 ± 0.10 ^cde^	8.00 ± 0.45 ^abcd^	7.90 ± 0.51 ^a^	ND	ND
	30	7.67 ± 0.17 ^abc^	7.83 ± 0.32 ^abcde^	7.43 ± 0.49 ^abcd^	ND	ND
	45	8.40 ± 0.32 ^a^	7.60 ± 0.49 ^bcde^	6.53 ± 0.61 ^bcd^	2.42 ± 0.18 ^b^	2.15 ± 0.31 ^c^
CB	1	6.53 ± 0.45 ^ef^	8.20 ± 0.16 ^abc^	7.50 ± 0.35 ^abc^	ND	ND
	15	7.13 ± 0.49 ^cdef^	7.96 ± 0.33 ^abcd^	7.50 ± 0.50 ^abc^	ND	ND
	30	7.55 ± 0.18 ^bcd^	7.63 ± 0.81 ^bcde^	7.46 ± 0.41 ^abc^	ND	ND
	45	8.10 ± 0.53 ^ab^	7.26 ± 0.20 ^de^	6.26 ± 0.55 ^cd^	ND	ND
CPB	1	6.70 ± 0.45 ^ef^	8.42 ± 0.23 ^ab^	7.63 ± 0.57 ^ab^	ND	ND
	15	6.84 ± 0.28 ^def^	8.50 ± 0.21 ^a^	7.60 ± 0.94 ^abc^	ND	ND
	30	7.49 ± 0.23 ^bcd^	8.27 ± 0.11 ^abc^	7.23 ± 1.10 ^abcd^	ND	ND
	45	8.40 ± 0.32 ^a^	7.46 ± 0.12 ^cde^	6.43 ± 0.47 ^bcd^	ND	ND

Data represented in means ± SD. ND represents the undetected data (*n* = 5; *p* ≤ 0.05). Microbial count was recorded as colony forming unit per milliliter (CFU/mL). Means that the same column with different superscript letters are significantly differences. CS: Control cheese; CP: Probiotic cheese MG847589; CB: Cheese manufactured with bacteriocin; CPB: Cheese manufactured with probiotics and bacteriocin.

**Table 3:** Texture evaluation for white cheese manufactured by various strategies.

Texture Parameter	Unit	CS	CP	CB	CPB
**Hardness cycle 1**	g	2648.73 ± 2.30 ^c^	3357.73 ± 6.80 ^b^	2525.73 ± 4.04 ^d^	3988.03 ± 6.00 ^a^
**Adhesive force**	g	156.02 ± 3.60 ^d^	378.17 ± 3.54 ^a^	200.02 ± 2.64 ^c^	220.02 ± 2.00 ^b^
**Adhesiveness**	mJ	1.60 ± 0.26 ^a^	4.53 ± 0.64 ^a^	2.00 ± 0.34 ^a^	3.53 ± 0.50 ^b^
**Hardness cycle 2**	g	2689.03 ± 2.64 ^b^	2833.03 ± 3.0 ^a^	2016.03 ± 4.58 ^d^	2555.33 ± 4.72 ^c^
**Hardness work cycle 2**	mJ	66.50 ± 5.63 b	74.50 ± 3.12 ^a^	56.16 ± 2.92 ^c^	60.00 ± 4.35 b^c^
**Springiness**	mm	6.44 ± 0.14 c	6.71 ± 0.18 ^ab^	7.30 ± 0.20 ^a^	6.91 ± 0.20 ^b^
**Springiness index**	-	0.92 ± 0.05 b	0.98 ± 0.03 ^ab^	1.09 ± 0.10 ^a^	1.09 ± 0.05 ^a^

For the same raw data, means with different superscript letters are significantly different (*p* ≤ 0.05). CS: Control cheese; CP: Probiotic cheese MG847589; CB: Cheese manufactured with bacteriocin; CPB: Cheese manufactured with probiotics and bacteriocin.

**Table 4:** Inhibitory effects of *Lactobacillus paracasei* MG847589 and/or bacteriocin, on aflatoxins (AFB_1_ or AFM_1_).

Cheese Treatments	Aflatoxin Reducing in Concentration (ng/mL) and the Reduction Ratio (%)					
	(2 h)		(24 h)		(48 h)	
Spiked cheese (AFB_1_)						
	**ng/mL**	**(%)**	**ng/mL**	**(%)**	**ng/mL**	**(%)**
CS	401.33 ± 0.47 ^a^	0	400 ± 1.63 ^a^	0	400.33 ± 0.94 ^a^	0
CP	212.66 ± 1.69 ^b^	47.01 ± 0.59 ^a^	193.33 ± 2.05 ^b^	51.66 ± 0.75 ^a^	148 ± 2.16 ^b^	63.0 ± 0.54 ^a^
CB	195.66 ± 0.94 ^c^	51.25 ± 0.42 ^b^	182 ± 1.41 ^c^	54.50 ± 0.48 ^b^	135.33 ± 1.69 ^c^	66.17 ± 0.21 ^b^
CPB	179.5 ± 0.5 ^d^	55.27 ± 0.71 ^c^	166 ± 0.1 ^d^	58.5 ± 0.47 ^c^	121.5 ± 2.5 ^d^	69.63 ± 0.44 ^c^
Spiked cheese (AFM_1_)						
	**ng/mL**	**(%)**	**ng/mL**	**(%)**	**ng/mL**	**(%)**
CS	405 ± 1.41 ^a^	0	404 ± 1.41 ^a^	0	404.33 ± 2.86 ^a^	0
CP	236.60 ± 1.69 ^b^	41.58 ± 0.50 ^a^	203.66 ± 2.35 ^b^	49.59 ± 0.93 ^a^	143 ± 1.41 ^b^	64.6 ± 0.70 ^a^
CB	209.33 ± 0.47 ^c^	48.31 ± 1.28 ^b^	192.66 ± 1.24 ^c^	52.31 ± 0.19 ^b^	133.33 ± 3.29 ^c^	66.99 ± 0.31 ^b^
CPB	179.66 ± 1.88 ^d^	55.64 ± 0.63 ^c^	160 ± 1.41 ^d^	60.39 ± 0.25 ^c^	115.66 ± 1.69 ^d^	71.38 ± 0.75 ^c^

Values of each column with the different superscript letter were significantly different (*n* = 3; *p* ≤ 0.05). CS: Control cheese; CP: Probiotic cheese MG847589; CB: Cheese manufactured with bacteriocin; CPB: Cheese manufactured with probiotics and bacteriocin.

**Table 5:** Treatments and inoculation levels of antimicrobial and anti-mycotoxigenic assays.

-ve Control	Food Pathogens			Mycotoxins	
*Lb. paracasei* MG847589	+*S. aureus*	+*A. parasiticus* ITEM 698	+*P. chrysogenum* ATCC 11709	+Aflatoxin B_1_	+Aflatoxin M_1_
**7 log_10_ CFU/g**	6.5 log_10_ CFU/g	5 log_10_ CFU/g	5 log_10_ CFU/g	400 ng/mL	400 ng/mL
**CS**	CSS	CSA	CSP	CSB_1_	CSM_1_
**CP**	CPS	CPA	CPP	CPB_1_	CPM_1_
**CB**	CBS	CBA	CBP	CBB_1_	CBM_1_
**CPB**	CPBS	CPBA	CPBP	CPBB_1_	CPBM_1_

Every treatment was inoculated individually to *Lb. paracasei* MG847589 and/or its bacteriocin.

**Table 6:** Components required for white cheese manufacturing.

Components	Quantity
**Skim Milk**	145 (g)
**Milk protein concentrate**	145 (g)
**Butter**	70 (g)
**Commercial stabilizer**	2.5 (mL)
**NaCl**	15 (g)
**CaCl_2_**	2 (g)
**Rennet (1%)**	10 (mL)
**H_2_O**	620 (mL)

## Data Availability

All data are available in the present manuscript.

## Supplementary Materials

The following supporting information can be downloaded at https://www.mdpi.com/article/10.3390/toxins16020093/s1, Figure S1:. Diagram shows the steps of manufacturing functional soft white cheese MPC, Milk protein concentrate; RCM, Reconstituted skimmed milk.
