# Supplementary material for: Biopreservative and Anti-Mycotoxigenic Potentials of Lactobacillus paracasei MG847589 and Its Bacteriocin in Soft White Cheese
Source: Toxins (Basel). 2024 Feb 7;16(2):93. doi: 10.3390/toxins16020093 (PMC10891891; doi:10.3390/toxins16020093)
Supplement: Supplementary file 1 [file toxins-16-00093-s001.zip › toxins-2590887-supplementary.pdf]

Biopreservative and Anti-Mycotoxigenic Potentials of *Lactobacillus paracasei* MG847589 and Its Bacteriocin in Soft White Cheese

Mohamed G. Shehata [1,2], Tawfiq Alsulami [3], Nourhan M. Abd El-Aziz [1], Hagar S. Abd-Rabou[1], Sobhy A. El Sohaimy [1,4], Amira M. G. Darwish [1], Karolina Hoppe [5], Hatem S. Ali [6] and Ahmed Noah Badr [7,*]

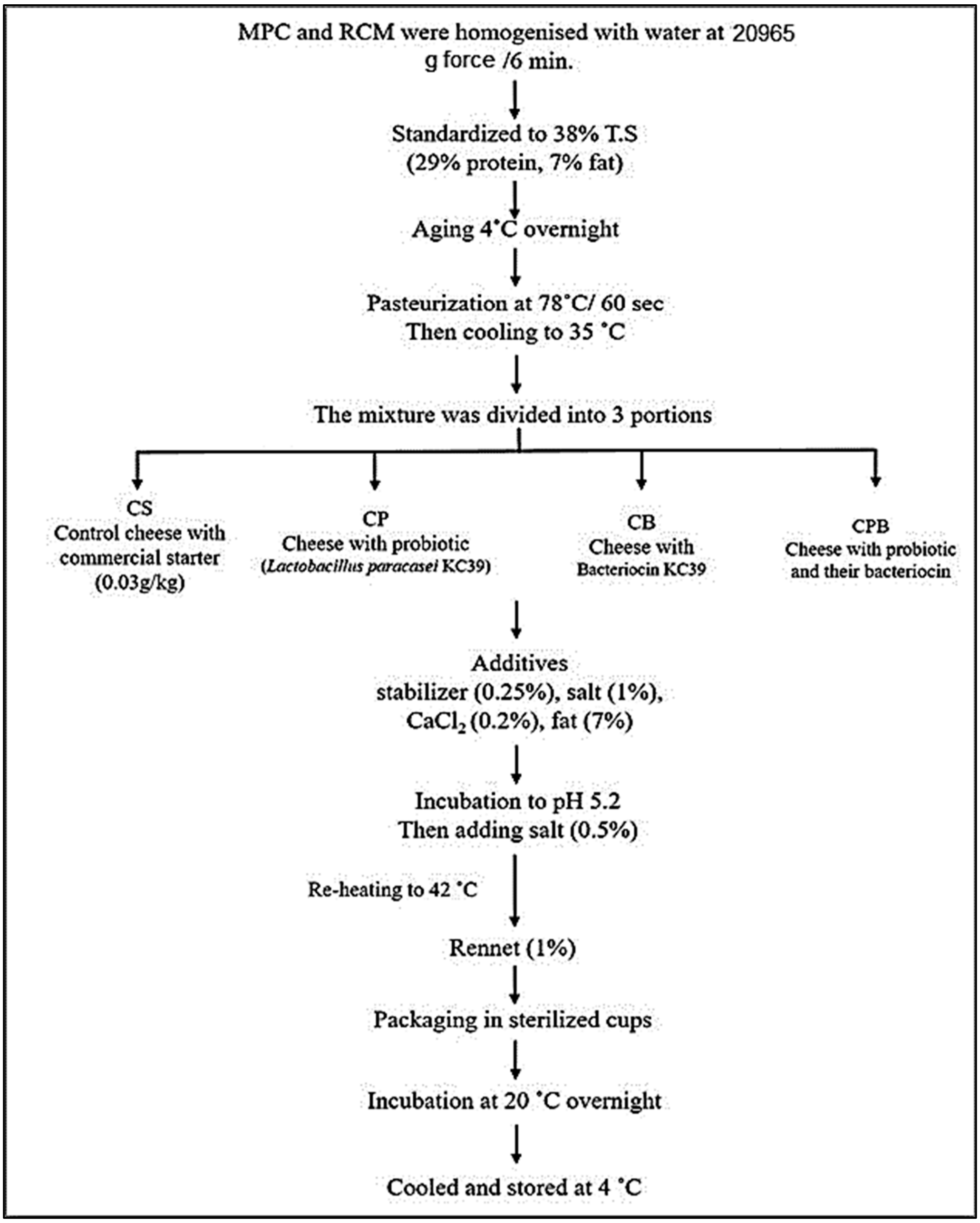

Figure S1. Diagram shows the steps of manufacturing functional soft white cheese MPC, Milk protein concentrate; RCM, Reconstituted skimmed milk.

Control cheese prepared with commercial starter (CS, 1.81× 10^9 CFU/mL), a probiotic cheese (CP; 1.34× 10^9 CFU/mL) of *L. paracasei* MG 847589, and a bacteriocin-supplemented cheese (CB; at 500 AU/mL).
